# Unraveling the Gordian knot: genetics and the troubled road to effective therapeutics for Alzheimer’s disease

**DOI:** 10.1093/genetics/iyab185

**Published:** 2021-10-28

**Authors:** Linda L Restifo

**Affiliations:** 1 Department of Neurology, University of Arizona Health Sciences, Tucson, AZ 85724, USA; 2 Department of Cellular and Molecular Medicine, University of Arizona Health Sciences, Tucson, AZ 85724, USA; 3 Department of Neuroscience and Graduate Interdisciplinary Program in Neuroscience, University of Arizona, Tucson, AZ 85721, USA; 4 Graduate Interdisciplinary Program in Genetics, University of Arizona, Tucson, AZ 85719, USA; 5 Evelyn F. McKnight Brain Institute, University of Arizona, Tucson, Arizona 85724, USA; 6 BIO5 Interdisciplinary Research Institute, University of Arizona, Tucson, AZ 85721, USA

**Keywords:** amyloid plaque, neurofibrillary tangle, tau, EOAD, LOAD, *APP*, *PSEN1*, *PSEN2*, *MAPT*, FDA

## Abstract

In the late 20th century, identification of the major protein components of amyloid plaques and neurofibrillary tangles provided a window into the molecular pathology of Alzheimer’s disease, ushering in an era of optimism that targeted therapeutics would soon follow. The amyloid-cascade hypothesis took hold very early, supported by discoveries that dominant mutations in *APP*, *PSEN1*, and *PSEN2* cause the very rare, early-onset, familial forms of the disease. However, in the past decade, a stunning series of failed Phase-3 clinical trials, testing anti-amyloid antibodies or processing-enzyme inhibitors, prompts the question, *What went wrong?* The FDA’s recent controversial approval of aducanumab, despite widespread concerns about efficacy and safety, only amplifies the question. The assumption that common, late-onset Alzheimer’s is a milder form of familial disease was not adequately questioned. The differential timing of discoveries, including blood–brain–barrier-penetrant tracers for imaging of plaques and tangles, made it easy to focus on amyloid. Furthermore, the neuropathology community initially implemented Alzheimer’s diagnostic criteria based on plaques only. The discovery that *MAPT* mutations cause frontotemporal dementia with tauopathy made it even easier to overlook the tangles in Alzheimer’s. Many important findings were simply ignored. The accepted mouse models did not predict the human clinical trials data. Given this lack of pharmacological validity, input from geneticists in collaboration with neuroscientists is needed to establish criteria for valid models of Alzheimer’s disease. More generally, scientists using genetic model organisms as whole-animal bioassays can contribute to building the pathogenesis network map of Alzheimer’s disease.

Those who cannot remember the past are condemned to repeat it.

*George Santayana, 1905 (often misattributed to Winston Churchill* ^1^ *)*

## Origins of this article

For nearly three decades, I have taught biomedical graduate students the science of neurological and other disorders, focusing on genetic factors that drive disease pathogenesis and on strategies for therapeutics discovery. Every five years or so, Alzheimer’s disease is the focus of a several-week module. Thus, my perspective is that of an educator, guiding students through a vast and somewhat contradictory terrain of published literature that is outside my first-hand research expertise. For the last decade, additional web resources, such as ClinicalTrials.gov, were often added to assignments of peer-reviewed papers. Early on, it seemed reasonable to reassure students that we would make sense of Alzheimer’s disease. More recently, however, the teaching challenge grew as biotech business news articles reported a series of failed Phase-3 clinical trials. 

Four years ago, a report card of sorts in *Bloomberg News* concluded that “Big Pharma is losing a fortune trying to cure Alzheimer’s” (Kresge and Bloomfield 2017; see Web Resources). This was echoed two years later in *Fortune* with the headline, “Alzheimer’s: A Trail of Disappointment for Big Pharma” (Mukherjee 2019; see Web Resources). Nonetheless, in early June 2021, the U.S. Food and Drug Administration (FDA) granted a highly controversial approval for an investigational biologic that critics say shows little evidence of improving cognition in patients with Alzheimer’s disease *and* has a high rate of serious adverse events (Carome 2021; see Web Resources). Thus, the primary educational challenge that I and other professors face is how to help aspiring scientists understand why, despite enormous investment and effort in basic and clinical research for the past four decades, there are still no safe-and-effective therapies that slow Alzheimer’s disease progression. As for explaining the FDA’s unexpected decision, that will have to await the investigations currently underway by several government agencies (see below).

I propose that discoveries in human genetics were misapplied to the pathogenesis of Alzheimer’s disease in a well-intentioned push to jump-start the discovery of therapeutic agents. Several key interacting factors were (1) the publication timeline of key scientific discoveries; (2) the Mendelian genetics of familial dementias; and (3) the assumptions that underlie drug-development efforts based on the amyloid-cascade hypothesis.

## Alzheimer’s disease poses questions and challenges

Alzheimer’s disease is an adult-onset, chronic, progressive condition characterized by cognitive decline, typically starting slowly and insidiously with short-term memory deficits. Many patients also manifest mood changes and dysfunctional behaviors that are difficult for caregivers to manage. Neurodegeneration eventually affects many cortical areas, causing gross atrophy, with prominent involvement of the most phylogenetically advanced regions, the neocortex. However, at the outset, it tends to show regional selectivity with cholinergic pathways and the hippocampus, an essential structure for memory formation, being particularly vulnerable. In many patients, hippocampal volume reduction can be detected by high-resolution MRI. That raises several big questions—*When does the disease process begin? How can disease pathogenesis be slowed or prevented?* But these are relatively modern late-20th-century questions that required better understanding of normal cognitive aging and enough cellular and molecular-level advances to support the very idea that Alzheimer’s disease could plausibly be treatable.

**Figure 1:**
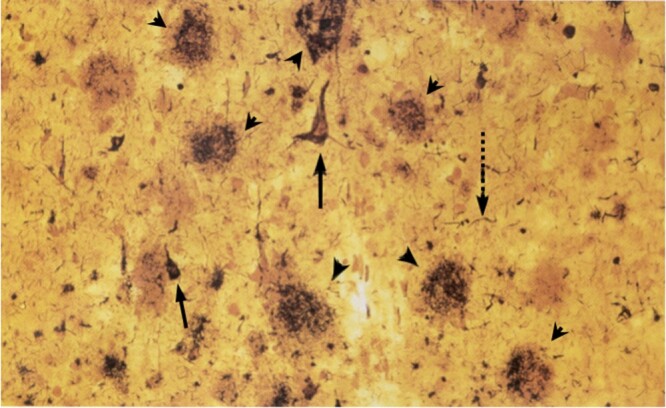
Plaques and tangles: the classic neuropathology of Alzheimer’s disease. Bielschowsky silver-stained section of cerebral cortex from a patient with Alzheimer’s disease. Reproduced and annotated with permission from Massachusetts Medical Society (Yankner and Mesulam, 1991). Amyloid plaques (arrowheads) are spherical, extracellular, insoluble deposits that involve multiple neurons and are often surrounded by dystrophic neurites. They contain small peptide fragments of APP that have assembled as amyloid fibrils. They increase with normal aging and may be cleared by microglia. They may be seen in large numbers in the brains of cognitively normal people. NFT (solid arrows) are also insoluble, but intracellular. Each tangle fills a single neuronal cell body and extends into the apical dendrite, creating a flame shape. They consist primarily of hyperphosphorylated microtubule-associated protein tau, assembled into paired helical filaments. When the neuron dies, the tangle remains. Tangle numbers and distribution correlate well with cognitive decline. A tau-containing neuropil thread (broken arrow) is also highlighted.

As with any neurodegenerative disease, the fundamental mystery is, *What is the agent of destruction that causes neuronal dysfunction and loss?* The first clues came from autopsy histopathology, notably the work of Aloysius “Alois” Alzheimer and other clinician-pathologists working in early 20th century. Using Bielschowsky silver staining, light microscopy and camera lucida technologies, Alzheimer and his contemporaries, including Oskar Fischer and Gheorghe Marinescu, drew two types of abnormal structures seen in the postmortem brain sections of their elderly demented patients (reproduced in Ryan *et al.* 2015; Broxmeyer 2017). “Senile plaques” are extracellular, circular Medusa-like arrays of tendrils surrounding a smooth center. (At the time, “senile” referred to old age; thus, senile dementia and presenile dementia distinguish age of onset.) A rough English translation of Alzheimer’s writing at the time would be, “Senile plaques have something to do with dementia.” He also described a second abnormality translated as, “peculiar fibrillary changes of the nerve cells,” later named neurofibrillary tangles (NFTs). These intracellular structures have a characteristic flame shape, filling the neuronal cell body, with the tapered end extending into the apical dendrite. These were two tantalizing smoking guns, albeit in end-stage autopsy brain tissue (Figure  1).

## Plaques *vs* tangles: a scientific rivalry fueled by genetics

As science became more mechanistic, the question evolved, *What are the bullets?* For some scientists, that question has been answered by focusing on plaques. First, Alzheimer’s senile plaques were revealed to contain “amyloid,” material with particular staining properties, now known to reflect filamentous protein aggregates with specific biophysical characteristics (Serpell 2014). Thus, senile plaques became “amyloid plaques.” The “amyloid cascade hypothesis” of Alzheimer’s disease pathogenesis (Hardy and Higgins 1992) was based on the isolation from plaques in the mid-1980s of a novel peptide. Named “amyloid-beta” or “A-beta” (Aβ), it is small (4-kD, 40–45 amino acids) with amyloidogenic properties. Moreover, in some lab assays, application of Aβ peptide to neuronal cultures caused cell death. Aβ deposition and amyloid plaque formation were proposed to be the primary triggers of neurodegeneration. This quickly became the dominant mechanistic viewpoint and a cornerstone of research on therapeutic strategies for Alzheimer’s disease.

It is worth noting that tissue deposits of amyloid are seen in many other diseases, often with monogenic etiology; in each amyloidosis, a specific protein folds abnormally to form amyloid fibrils (Pande and Srivastava 2019). Aβ is derived by abnormal processing from a much larger, previously unknown protein. While it is not clear who first applied the name “amyloid precursor protein” (APP) to the parent molecule of Aβ and the gene encoding it (possibly NCBI, the National Center for Biotechnology Information), within a year of its discovery that moniker came into common use, initially preceded by β (*e.g.*, Selkoe *et al.* 1988). Because of this nomenclature decision, along with the prominence of Alzheimer’s disease research publications and the urgency about its increasing prevalence in the aging population, the word “amyloid” has become highly associated with the disease.

Meanwhile, NFTs were shown by electron microscopy to contain paired helical filaments (PHF), *i.e.*, different from the fibrils in amyloid plaques. Immunostaining methods were used to identify microtubule-associated protein tau (MAPT) as the main component of PHF, and to suggest that this tau was abnormal due to hyperphosphorylation. But there was controversy about the specificity of some of the antibody reagents, and it took another five years to verify these findings with biochemical methods (Kosik 1990).

Instead of focusing on amyloid plaques *and* PHF-containing tangles, with the pair of them viewed as a unique duo of pathological hallmarks that defined Alzheimer’s disease [as in the more recent analysis by Nelson *et al.* (2009)], there instead emerged a battle *between* them, more akin to a sports rivalry than to objective scientific debate. For three decades, team amyloid won the battle, but perhaps the playing field was not even. Writing in *STAT*, an online periodical focused on science and medicine, and based on numerous interviews with the researchers involved, experienced science journalist Sharon Begley referred to amyloid proponents as a “cabal” (Begley 2019; see Web Resources). This view was reinforced in an impassioned but more traditional comprehensive review article (Mullane and Williams 2020). What was the battle for?—scientific bragging rights, as well as research funding—those were the obvious prizes with implications for individual professional advancement. Ultimately, however, it was a battle over the very definition of the disease.

For more than two decades, American neuropathologists making an autopsy-based diagnosis of Alzheimer’s disease counted amyloid plaques in specific brain regions and adjusted for the patient’s age to derive an age-adjusted plaque score (Khachaturian 1985; Mirra *et al.* 1993). Why? Because amyloid plaques accumulate with normal aging. In addition, pathologists would not make the diagnosis without a clinical history of dementia. Why? Because occasionally, the brain of a cognitively normal person has lots of plaques. In other words, the specificity of amyloid plaques as a solo diagnostic marker of Alzheimer’s disease was somewhat problematic from the start. In contrast, NFT numbers and distribution provide a better pathological correlate with clinical disease (Braak and Braak 1991). Despite this, tangles were initially excluded from the neuropathological diagnostic criteria for Alzheimer’s disease. Hence, clinicians and scientists came to equate Alzheimer’s disease with amyloid plaques even though they were aware of tangles. How could such an unbalanced view of such an important topic become entrenched? While science “politics” played a role (Mullane and Williams 2020) the mis-application of human genetics was part of the problem.

A series of genetics discoveries strongly reinforced the focus on Aβ and amyloid plaques (Tanzi *et al.* 1996). First, in 1987, the *APP* gene (MIM# 104760) was mapped to chromosome 21, three copies of which cause Down syndrome (OMIM, Online Mendelian Inheritance in Man; see Web Resources). When patients survive to middle adulthood (which became increasingly common with improved medical and surgical care), they are at high risk for early-onset Alzheimer’s disease (EOAD), with abundant amyloid plaque deposition seen at autopsy. This suggested that too much APP is bad, and supported the view that amyloid drives the disease mechanism. Second, there are small numbers of families in which EOAD is transmitted as a dominant disorder. In 1991 came the first report of a family with *APP* mutations linked to EOAD. Later, other *APP* mutations were identified in families around the world, including many different missense alleles, but also whole-gene duplications and changes in regulatory sequences that increase gene expression—pointing to a gain-of-function genetic mechanism. This did not explain all familial clusters. In 1995, two new genes were reported with dominant mutations causing EOAD. Because the functions of these genes were not previously known, they were named “presenilin” 1 and 2, referring to the *early* onset of disease (*i.e.*, presenile). *PSEN1* (MIM# 104311) and *PSEN2* (MIM# 600759) encode subunits of γ-secretase, an enzyme that processes APP by endoproteolytic cleavage within the transmembrane domain (Kimberly and Wolfe 2003).

Familial Alzheimer’s disease (FAD)-causing mutations in *APP*, *PSEN1*, and *PSEN2* increase the production of amyloidogenic Aβ peptides. In contrast, the genetics of *MAPT* (MIM# 157140) did not connect to Alzheimer’s disease. Rather, a clinically distinct familial disorder, frontotemporal dementia (FTD) with parkinsonism, was mapped to dominant mutations in *MAPT* (Dumanchin *et al.* 1998). The postmortem neuropathology of FTD includes NFT and other deposits containing abnormal tau in neurons and glia, leading to the term “tauopathy.”

Of course, Alzheimer’s disease is also a tauopathy. However, the genetics of FAD pointed to amyloid production as the proximate cause, while the genetics of tau pointed away from Alzheimer’s disease. Thus, even as members of the neuropathology community began writing about incorporating NFT into postmortem analyses (National Institute on Aging and Reagan Institute Working Group 1997), the amyloid cascade hypothesis had already become deeply ingrained. Few questioned the relationship between early- and late-onset disease or the prominent vascular phenotype, cerebral amyloid angiopathy, seen with some *APP* mutations. Instead, the amyloid cascade hypothesis led to two main therapeutic strategies in academic and industry research labs; both strategies were reliant on mouse models for preclinical studies. The first approach is to use monoclonal antibodies to bind and clear amyloid (the names of these biologic agents end in “umab”). The second is to reduce amyloid production by inhibiting β-secretase, BACE (the names of these small-molecule drugs end in “cestat”). BACE activity generates the extracellular cleavage needed to produce Aβ.

The next major breakthrough was development of a novel brain-imaging biomarker. A radiolabeled thioflavin-T analog, ^11^C-PiB (often called “the Pittsburgh compound”), crosses the blood-brain barrier and binds selectively to fibrillar Aβ, thereby allowing visualization in living people of amyloid plaque location and quantity (Johnson *et al.* 2007). This permits patients to be screened by positron emission tomography (PET) brain imaging prior to enrollment in clinical trials, as well as to undergo follow-up scans to determine if amyloid-plaque burden has been reduced by an investigational agent. Efforts to find a comparable ligand for hyperphosphorylated tau took more than an additional decade (Tagai *et al.* 2021).

## The clinical-trials debacle demands re-evaluation of the anti-amyloid strategy

By the time the neuropathology community implemented a systematic plaques-and-tangles scoring rubric for the postmortem diagnosis of Alzheimer’s disease (Montine *et al.* 2012), the amyloid-targeted clinical trials pipeline was already in full gear. In fact, the failures were just starting to emerge. In 2012, administration of bapineuzumab to subjects with mild-to-moderate Alzheimer’s disease failed in a Phase-3 trial, a collaboration between Johnson and Johnson and Pfizer (Carroll 2012; see Web Resources). Several years later, another anti-amyloid antibody, Eli Lilly’s solanezumab, failed in mild Alzheimer’s disease (Loftus 2016; see Web Resources). In 2019, Roche’s crenezumab failed in two Phase-3 trials treating subjects with prodromal or mild Alzheimer’s disease (Pagliarulo 2019; see Web Resources). Moreover, it wasn’t just the anti-amyloid antibodies; the BACE inhibitors were in trouble as well (Keown 2018; see Web Resources), with three of them failing due to lack of efficacy (Eli Lilly’s lanabecestat) or serious toxicity concerns (Merck’s verubecestat and J&J/Janssen’s atabacestat). Some of the investigational agents had been shown to reduce amyloid plaque burden in subjects’ brains, but this was not accompanied by reduced rates of cognitive decline. In other words, the agents were working just as had been intended, but they did not slow Alzheimer’s disease progression.

Amyloid clearance was also seen with aducanumab, Biogen and Eisai’s antibody, which was touted as better than the others because it targets oligomeric forms of Aβ, *i.e.*, the precursors to the larger aggregates. Despite this seemingly promising feature, aducanumab failed a “futility analysis” performed midway through two Phase-3 clinical trials (Serwick 2019; see Web Resources). Later that year, Biogen announced it was scrapping the program (Maddipatla 2019; see Web Resources). In a commentary for *Science*, a medicinal chemist with considerable biotech-industry experience wrote, “Amyloid definitely has something to do with Alzheimer’s disease,” (Lowe 2019; see Web Resources) echoing Alzheimer’s words from over a century ago. This is not funny.

## Root cause analysis

With drug discovery and development, the hope is that positive results in preclinical research, especially the work conducted on animal models of disease, and early-stage clinical trials will predict therapeutic efficacy in large, expensive Phase-3 clinical trials. (Safety will often be harder to predict.) When there are repeated failures at Phase 3, across an industry and with related therapeutic strategies, the need for reassessment seems obvious. But, unlike with major accidents investigated by the National Transportation Safety Board, it is not clear what agencies will mandate a root-cause analysis of the failed Alzheimer’s trials, let alone recommend guidelines for future research. Nor is there any certainty that the data will become available for independent scientific scrutiny beyond the FDA-mandated reporting of summary results on ClinicalTrials.gov. Surely the senior leadership of the National Institutes of Aging (NIA) and of Neurological Disorders and Stroke (NINDS), which support basic and clinical research efforts in “the search to find treatment and prevention strategies” (NIH Research on Alzheimer’s disease and related dementias; see Web Resources), will have much to say about the past and the future.

Proponents of the amyloid-cascade hypothesis would suggest several potential sources of error that could plausibly explain why targeting amyloid has not been successful despite, in their view, the hypothesis being fundamentally correct (Table  1). Some of these are common to many failed therapeutic trials—perhaps the dosage or route of administration were not optimal. Perhaps the cognitive tests used as outcome measures lack some combination of sensitivity and specificity. Unrecognized differences in placebo-response genetics between treatment and placebo arms could also contribute to false-negative clinical trial failures (Hall *et al.* 2015). Finally, as with other chronic diseases, if pathogenesis begins much earlier in life, it is possible that mild clinical disease, or even minimal cognitive impairment (MCI; Lindeboom and Weinstein 2004), may already be too late for effective intervention to slow progression. In that case, prevention may be the better strategy (Vina and Sanz-Ros 2018), but very challenging for clinical trials design especially in terms of duration.

**Table 1:** Plausible explanations for failures of clinical trials based on the amyloid-cascade hypothesis of Alzheimer’s disease

**A. Generic issues that plague many clinical trials**	**Possible consequences**
• Suboptimal dosage, duration, regimen, or route of administration	> Evidence of therapeutic benefit may have been missed
• Suboptimal cognitive-test designs as outcome measures	> Tests may not reveal clinically meaningful improvement
• Arms mis-matched for genotypes regulating placebo response	> Slower-than-normal cognitive decline in placebo arm
• Disease process begins decades earlier than detected	> Even MCI may be too late to treat, prevention a better goal
**B. Specific issues related to Alzheimer's disease**	**Possible consequences**
• Pathogenic form(s) of Ab peptide not correctly identified	> Therapeutic antibodies have the ‘wrong’ amyloid target
• Flawed animal models	> Mouse models failed to predict lack of clinical efficacy
• Lack of specific, sensitive peripheral biomarkers	> No simple leading indicators to guide predictions of success
• Excessive LOAD patient heterogeneity in clinical trials	> LOAD with multiple pathogenesis subtypes mixed together
• Assumption that LOAD has same mechanism as FAD, just slower	> Anti-amyloid strategy may only work in familial cases

More connected to the amyloid-clearing strategy, perhaps the investigational antibodies target the ‘wrong’ molecular species of amyloid peptide. Recent data have revealed a larger and more dynamic set of peptide-size classes resulting from APP processing by γ-secretase (Wolfe 2012). It remains uncertain which one(s), or their ratios, are initiators and/or drivers of disease pathogenesis. Similarly, immunostaining, which has higher sensitivity than traditional histopathological methods, has revealed several types of amyloid-containing brain lesions, such as diffuse plaques. The potential pathological roles of these molecular and cellular varieties of Aβ, including soluble and oligomeric species, remain to be clarified. It is also possible that the damaging effects of Aβ are on cerebral vasculature (Malek-Ahmadi *et al.* 2021), long before its accumulation in the brain parenchyma is evident.

With 20-20 hindsight, there were four key vulnerabilities that pervaded the industry-led, amyloid-focused therapeutic programs (Table  1). First, the mouse genetic models (Puzzo *et al.* 2015) used in academia and industry, lack validity based on common-sense standards well-described by Nestler and Hyman (2010), notably for neuropsychiatric disorders. Mice are short-lived lower mammals, with very small volumes of neocortex, the site of much Alzheimer’s disease pathology. It was very difficult to engineer mice that developed plaques and tangles. That breakthrough, the “triple transgenic” mouse model (Oddo *et al.* 2003), expresses three dominant mutations, two (in *APP* and *PSEN1*) that *individually* cause Alzheimer’s disease *plus* one (in *MAPT*) that causes FTD. To date, no mouse model shows extensive neurodegeneration comparable to that seen in the human disease. Thus, mouse genetic models do not have face or construct validity for Alzheimer’s disease. Given those limitations, perhaps it is not surprising that mouse models did not have predictive pharmacological validity for therapeutic interventions in humans. Nonetheless, recent systematic investigation of genetic background effects on behavioral and pathological phenotypes of the FAD5X mouse model are providing biological insights as well as a new strain with better similarity to late-onset Alzheimer’s disease (LOAD) transcriptome profiles (Neuner *et al.* 2019). Moreover, the MODEL-AD Consortium was formed with the explicit goal of developing, validating, and distributing better mouse models of LOAD (Oblak *et al.* 2020). Studying genetic background effects in improved mouse models may help make sense of the genetic complexity and heterogeneity of LOAD, which brings us to the second vulnerability of the clinical trials.

The study participants may have been more heterogeneous than appreciated by the investigators. For example, in a recent meta-analysis, Ferreira *et al.* (2020) demonstrated that non-familial LOAD can be partitioned into four subtypes, of which “typical” is only a bit more than half; the other half is divided among several categories with different clinical and pathological profiles, but not so distinct that they would be obvious when enrolling patients in clinical trials focused on MCI or mild Alzheimer’s disease. These results were reinforced by an independent study based on patterns of tau neuropathology observed by PET imaging (Vogel *et al.* 2021). Other investigators have associated clinical heterogeneity in Alzheimer’s disease with genetic differences, including *APOE* (MIM# 107741) genotype (Mukherjee *et al.* 2020). Deep dives into molecular-level heterogeneity have identified distinct transcriptome patterns among LOAD brains at autopsy, with associations to polymorphic variants that may modify disease risk (Milind *et al.* 2020) and aligning with differences among mouse models (Neff *et al.* 2021). Unrecognized Alzheimer’s disease heterogeneity could contribute to clinical trials failures in several ways, especially if treatment and placebo arms do not contain comparable groups of patients.

The third vulnerability was the lack of one or more validated, specific, and sensitive peripheral biomarkers of Alzheimer’s disease activity. Companies may well have thought this was not necessary once they could use PET imaging with the Pittsburgh compound to visualize amyloid-plaque burden. Peripheral biomarkers (ideally blood, but cerebrospinal fluid (CSF), might be required) are a big missing puzzle piece that could help identify individuals at risk for LOAD prior to any cognitive effects, as well as to inform both natural history studies (*e.g.*, the placebo arms of clinical trial) and responses to investigational therapeutics. Recent discoveries have focused on non-amyloid, non-tau proteins in plasma (Lindbohm *et al.* 2021). Regardless of how technologically simple or complex the biomarker is, until one knows that it reflects and/or predicts cognitive parameters that are clinically meaningful and, ideally, relevant to patients and caregivers, then the biomarker won’t be useful as a leading indicator of therapeutic success. Also unknown is whether clinically useful biomarkers will need to be specific to LOAD (as opposed to other dementias), or even to particular subtypes of LOAD.

**Figure 2:**
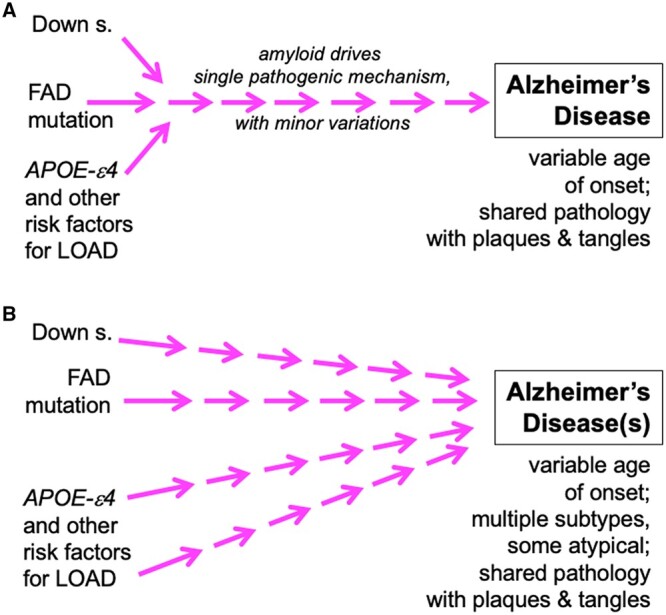
Diagrammatic representations of two alternative conceptual views of Alzheimer’s disease pathogenesis. Although not shown, *APOE4* genotype can influence Alzheimer’s disease risk and age of onset in Down syndrome and FAD. (A) The predominant single-mechanism view, with amyloid production and deposition driving disease pathogenesis. Late-stage therapeutic development has been based on viewing common LOAD as a slower form of FAD. (B) Alternative view, with multiple convergent mechanisms leading to “Alzheimer’s disease,” which has distinct subtypes. Factors that could be driving disease pathogenesis include inflammation, infection, co-existing cerebrovascular disease, and RNA modification causing interference with protein synthesis. The “true” diagram is more likely to be a highly branched network, rather than linear pathways, with interconnections and feedback loops. FAD, familial Alzheimer’s disease; LOAD, late-onset Alzheimer’s disease; s., syndrome.

The fourth and possibly most significant vulnerability was reliance on the assumption, based on shared autopsy neuropathology, that common LOAD arises by the same pathogenic mechanism, with minor variations and slower speed, as the very rare (≤1%) familial EOAD (Figure  2). This reasoning assumes that a fully penetrant monogenic disorder arises by the same pathway as late-onset disease controlled by polygenic and environmental influences (*e.g., APOE* genotype and head trauma, respectively). Yet, two to three decades separate the average age of onset of EOAD from the *beginning* of the age distribution of late-onset disease, which starts at ∼65 years with the incidence rising 10-fold over 20 years (Mayeux and Stern 2012). At the very least, the same-mechanism assumption needs to be investigated.

## Anti-amyloid therapy for prevention of Familial Alzheimer’s Disease

Perhaps the best test to date of the amyloid-cascade hypothesis is an ongoing Phase II clinical trial focused on healthy adults at risk for familial EOAD (ClinicalTrials.gov identifier NCT01998841; see Web Resources). Specifically, these are members of a very large family in Colombia, many of whom carry a *PSEN1* missense mutation (E280A). Designed as a prospective, longitudinal prevention trial, this double-blind study has enrolled presymptomatic adults and randomized those carrying the E280A mutation to crenezumab or placebo (Tariot *et al.* 2018). All mutation-negative subjects receive the placebo. The follow-up period is a minimum of 5 years, with an estimated study completion date of February 2022. Of course, if the specificity of the crenezumab antibody is ‘wrong’, this trial might fail to delay disease onset even if the underlying amyloid hypothesis is correct for familial EOAD.

## The attempted resurrection of aducanumab

A remarkable dynamic is playing out between Biogen, the FDA, and important stakeholders (Terry 2021; see Web Resources). After announcing in 2019 that it was pulling the plug on aducanumab, Biogen undertook additional data analysis. In 2020, they reported that, by focusing on data from the high-dose subgroups, they now believed that subjects who had received the highest dose seemed to be getting some benefit, but only in one of two parallel Phase-3 trials. When shown the new analyses, the FDA initially seemed most concerned about safety of the high dose, especially the risk of amyloid-related imaging abnormality-edema (ARIA-E), which can progress to brain hemorrhage. To clarify the safety risks, the FDA allowed a modified trial to move forward (Clinical Trials.gov identifier NCT04241068; see Web Resources). It was no longer randomized, double-blind, and placebo-controlled, but rather open-label with no placebo group. Despite the fact that this safety study will not be completed until 2023, in Fall 2020, the FDA allowed Biogen to submit a request for approval of aducanumab based on the high-dose data. The FDA could have required additional safety data first.

In November 2020, a panel of eleven independent experts, the FDA’s Peripheral and Central Nervous System Drugs Advisory Committee (a/k/a Adcom), reviewed Biogen’s data and voted against FDA approval of aducanumab (Marchione and Perrone 2020; see Web Resources). The independent panel raised concerns about both Biogen’s and the FDA’s reports, because the level of enthusiasm did not match the results of the complex statistical analyses (Brennan 2021; see Web Resources). Meanwhile, Alzheimer’s advocacy groups weighed in with letters to the FDA supporting approval of aducanumab, reminiscent of amicus briefs submitted to the Supreme Court (Alzheimer’s Association 2020, and UsAgainstAlzheimer’s 2020; see Web Resources). In contrast, a watchdog agency called for an investigation into the “inappropriate” relationship between the FDA and Biogen (Public Citizen 2020; see Web Resources).

In June 2021, the FDA granted Biogen conditional accelerated approval of aducanumab, trade name Aduhelm™, for Alzheimer’s disease. The decision was based on the data showing reduction of the amyloid-PET surrogate biomarker, rather than on clinical efficacy, and included the stipulation that the company conduct a Phase-4 (*i.e.*, post-marketing) randomized, placebo-controlled trial (FDA 2021; see Web Resources). Such conditional approval would be withdrawn if the therapeutic agent fails to demonstrate efficacy or if risk of ARIA-E or other serious adverse events is deemed too high. Of course, the FDA could have required an additional clinical trial first along with the safety data currently being collected. Instead, they allowed aducanumab to be marketed for up to nine years while the new Phase-4 data are collected. Biogen set the annual cost of treatment at about $56,000, well above the $10,000 that business analysts had been expecting and in the price ballpark of a curative treatment for hepatitis C (Armstrong 2021; see Web Resources). Until, the Centers for Medicare and Medicaid Services (CMS; see Web Resources) complete their analysis and determine whether and for whom aducanumab will be covered, it is difficult to estimate how many patients will receive the treatment.

Reaction was fast and furious, based on the dual controversies of science and procedures. The FDA’s Adcom members, three of whom resigned in protest, characterized the FDA decision as being “at odds with the evidence and with the agency’s biostatistical review” (Alexander *et al.* 2021). The Veteran’s Administration Health Care System declined to add aducanumab to its formulary based on efficacy and safety concerns (Fuller 2021 and Kansteiner 2021; see Web Resources). Several prominent academic medical centers have announced they will not administer aducanumab infusions at their clinical sites (Belluck July 2021; see Web Resources). Investigations are underway by two U.S. Congressional committees (Belluck September 2021; see Web Resources), the Office of Inspector General of the U.S. Department of Health and Human Services (see Web Resources), as well as by FDA itself (Robbins 2021; see Web Resources). Biogen submitted the aducanumab data to *JAMA* for publication, but after peer reviewers made requests for major changes, they withdrew the submission rather than comply or risk outright rejection (Herman 2021; see Web Resources).

In trying to make sense of the FDA approval, which has been greeted with negative reactions by both researchers (Mullard 2021) and clinicians (Silverman 2021; see Web Resources), perhaps we need input from social scientists. Senior FDA leaders, writing in an Op-Ed article to respond to widespread criticism, noted that they were influenced by input from many people affected by Alzheimer’s disease—patients, loved ones, and caregivers. “They made it clear that they wanted access to a treatment option with the *potential* to stop or delay their disease, and that they were *willing to accept some degree of uncertainty* [emphasis mine].” (Cavazzoni *et al.* 2021; see Web Resources). In the opening line of their reaction to the approval of aducanumab, the editorial board of a major business newspaper wrote, “The Food and Drug Administration gave hope to millions of Americans suffering from Alzheimer’s disease…” (*Wall Street Journal* 2021; see Web Resources). After four decades of intensive research, the desire for hope is understandable. The question remains whether people have been given false hope. Furthermore, is providing hope a primary responsibility of the national regulatory agency charged with evaluating efficacy and safety? And has the FDA, inadvertently perhaps, given the amyloid cascade hypothesis its seal of approval?

## The path forward: is the glass half full or half empty?

For now, let’s set aside the FDA’s stunning decision, except to acknowledge the dilemma it may pose for research mentors. The next time you tell your graduate students that they can’t cherry-pick their data, don’t be surprised by the response, “But that’s what happened with aducanumab, so why can’t we do it?” And let’s acknowledge that ‘filling the glass’ began in the early 1980s with the shift away from the assumption that LOAD could not be avoided, understood, or treated (Khachaturian 1984). Much has been learned, as demonstrated by an exponential rise since then of publications about Alzheimer’s disease and about healthy brain aging, with the prospect that those insights could be harnessed for prevention or treatment. Where one falls on the optimism scale probably depends a lot on one’s personal temperament. It remains uncertain whether the retrospective viewpoint—working backwards from the plaques and tangles of terminal pathology—holds the key to understanding Alzheimer’s disease. Moving forward, one hope is that the scientific community will not repeat or promote some of the problems of the past, *e.g.*, premature adoption of one hypothesis as dogma or fixation on specific research technologies without ensuring that the data generated can be connected to meaningful clinical parameters.

The biggest conceptual gap is the lack of a specific mechanistic pathway or, more likely, network diagram that explains pathogenesis *and* identifies the rate-limiting steps or drivers. This includes understanding the sequence of cellular ‘symptoms’ upstream of neuronal death—axonal transport? synaptic function? general metabolism? macromolecular synthesis? Until then, the various efforts underway may necessarily resemble a group of blind people exploring an elephant. The biggest missing preclinical tool is one or more valid Alzheimer’s disease models, which will require discussion across disciplines, especially genetics and neuroscience, to define validation criteria. Parallel approaches using higher mammals and patient-derived induced pluripotent stem cell lines each have strong appeal. Engineered genetic model organisms may be most valuable as whole-animal bioassays for a molecule or pathway.

While the renewed interest in tau is certainly welcome, quick or easy answers are unlikely. It remains unclear which of several species of hyperphosphorylated tau should be viewed as the pathogenic agent(s) (Wegmann *et al.* 2021). Just months ago, a Phase-2 clinical trial of a monoclonal antibody, gosuranemab, against extracellular *N*-terminal fragments of tau (eTau) in patients with MCI or mild Alzheimer’s disease failed on all clinical endpoints even though it reduced CSF levels of the target (Carroll 2021; see Web Resources). Perhaps eTau is the wrong target. It is also worth remembering that better correlation with disease progression does not necessarily mean causation. In the meantime, data from a recent paper identified an intriguing novel potential pathogenic mechanism for tau oligomers, namely interference with protein synthesis via interaction with methylated RNA transcripts (Jiang *et al.* 2021).

The role of inflammation and its molecular and genetic mediators in Alzheimer’s pathogenesis is receiving heightened attention, with microglia and astrocytes of key interest (*e.g.*, Forloni and Balducci 2018; Monterey *et al.* 2021). Inflammation may also help explain ARIA-E, because Aβ-antibody complexes promote neuroinflammation by microglial activation (Trudler *et al.* 2021). Biotech companies are considering the special challenges of targeting microglial activation in the CNS with drugs (Biber *et al.* 2019). Perhaps connected via inflammation, infection may be a driver of Alzheimer’s disease (Broxmeyer 2017; Ou *et al.* 2020). Of particular interest, because of available drugs and vaccines that prevent viral reactivation, are infections caused by neurotropic alphaherpesviruses (VZV, HSV-1, and HSV-2).

On the genetics front, two areas stand out as high-priority goals. The old one is to solve the enigma of *APOE* genotype variation, the most potent single-gene risk modifier of LOAD (Koutsodendris *et al.* 2021). The new one is to explore the contribution of somatic mutations in brain to Alzheimer’s and other neurodegenerative diseases (Miller *et al.* 2021).

With no obvious preventive or therapeutic agents “right around the corner,” what can be done while we await a better mechanistic understanding of Alzheimer’s disease origins and progression? There are two promising avenues with practical implications. One is to treat hyperexcitability in the LOAD brain, which may manifest as seizures or as subclinical epileptiform activity on EEG or magnetoencephalography (Kazim *et al.* 2021). Based on positive results in patients with MCI, a Phase-2 randomized, placebo-controlled clinical trial is underway to determine if levetiracetam, a commonly used antiepileptic drug, improves memory in patients with mild–moderate LOAD (Sen *et al.* 2021).

The other is to address the well-established clinical and pathological overlap between cerebrovascular disease and LOAD. They may simply co-occur in large numbers of elderly patients, but there may also be synergistic interactions between their pathogenic mechanisms, especially in the preclinical phase of LOAD (Malek-Ahmadi *et al.* 2021). The connections are strong enough that a large international group has called for heightened stroke-prevention efforts as a means of decreasing dementia risk (Hachinski *et al.* 2019).

## Footnotes

1
Walters 2016; see Web Resources.

## References

[iyab185-B1] Alexander GC , Knopman DS, Emerson SS, Ovbiagele B, Kryscio RJ, et al 2021. Revisiting FDA approval of aducanumab. N Engl J Med. 385:769–771. 34320282 10.1056/NEJMp2110468 PMC11694499

[iyab185-B2] Biber K , Bhattacharya A, Campbell BM, Piro JR, Rohe M, et al 2019. Microglial drug targets in AD: opportunities and challenges in drug discovery and development. Front Pharmacol. 10:840. 31507408 10.3389/fphar.2019.00840 PMC6716448

[iyab185-B3] Braak H , Braak E. 1991. Neuropathological stageing of Alzheimer-related changes. Acta Neuropathol. 82:239–259. 1759558 10.1007/BF00308809

[iyab185-B4] Broxmeyer L. 2017. Are the infectious roots of Alzheimer’s buried deep in the past? J Mol Path Epidemol. 2(S2.2):1–19.

[iyab185-B5] Dumanchin C , Camuzat A, Campion D, Verpillat P, Hannequin D, et al 1998. Segregation of a missense mutation in the microtubule-associated protein tau gene with familial frontotemporal dementia and parkinsonism. Hum Mol Genet. 7:1825–1829. 9736786 10.1093/hmg/7.11.1825

[iyab185-B6] Ferreira D , Nordberg A, Westman E. 2020. Biological subtypes of Alzheimer disease: a systematic review and meta-analysis. Neurology. 94:436–448. 32047067 10.1212/WNL.0000000000009058 PMC7238917

[iyab185-B7] Forloni G , Balducci C. 2018. Alzheimer’s disease, oligomers, and inflammation. J Alzheimers Dis. 62:1261–1276. 29562537 10.3233/JAD-170819 PMC5869993

[iyab185-B8] Hachinski V , Einhäupl K, Ganten D, Alladi S, Brayne C, et al 2019. Preventing dementia by preventing stroke: the Berlin Manifesto. Alzheimers Dement. 15:961–984. 31327392 10.1016/j.jalz.2019.06.001 PMC7001744

[iyab185-B9] Hall KT , Loscalzo J, Kaptchuk TJ. 2015. Genetics and the placebo effect: the placebome. Trends Mol Med. 21:285–294. 25883069 10.1016/j.molmed.2015.02.009 PMC4573548

[iyab185-B10] Hardy JA , Higgins GA. 1992. Alzheimer’s disease: the amyloid cascade hypothesis. Science. 256:184–185. 1566067 10.1126/science.1566067

[iyab185-B11] Jiang L , Lin W, Zhang C, Ash PEA, Verma M, et al 2021. Interaction of tau with HNRNPA2B1 and N(6)-methyladenosine RNA mediates the progression of tauopathy. Mol Cell. 81:4209–4227.e12. 34453888 10.1016/j.molcel.2021.07.038 PMC8541906

[iyab185-B12] Johnson KA , Gregas M, Becker JA, Kinnecom C, Salat DH, et al 2007. Imaging of amyloid burden and distribution in cerebral amyloid angiopathy. Ann Neurol. 62:229–234. 17683091 10.1002/ana.21164

[iyab185-B13] Kazim SF , Seo JH, Bianchi R, Larson CS, Sharma A, et al 2021. Neuronal network excitability in Alzheimer’s disease: the puzzle of similar versus divergent roles of amyloid beta and tau. eNeuro. 8: ENEURO.0418-20.2020. 10.1523/ENEURO.0418-20.2020 PMC8174042 33741601

[iyab185-B14] Khachaturian ZS. 1984. Scientific challenges and opportunities related to Alzheimer’s disease. Clin Pharm. 3:522–523. 6149032

[iyab185-B15] Khachaturian ZS. 1985. Diagnosis of Alzheimer’s disease. Arch Neurol. 42:1097–1105. 2864910 10.1001/archneur.1985.04060100083029

[iyab185-B16] Kimberly WT , Wolfe MS. 2003. Identity and function of gamma-secretase. J Neurosci Res. 74:353–360. 14598311 10.1002/jnr.10736

[iyab185-B17] Kosik KS. 1990. Tau protein and neurodegeneration. Mol Neurobiol. 4:171–179. 2135393 10.1007/BF02780339

[iyab185-B18] Koutsodendris N , Nelson MR, Rao A, Huang Y. 2021. Apolipoprotein E and Alzheimer’s disease: findings, hypotheses, and potential mechanisms. Annu Rev Pathol. doi: 10.1146/annurev-pathmechdis-030421-112756.Online ahead of print. 10.1146/annurev-pathmechdis-030421-112756 34460318

[iyab185-B19] Lindbohm JV , Mars N, Walker KA, Singh-Manoux A, Livingston G, et al 2021. Plasma proteins, cognitive decline, and 20-year risk of dementia in the Whitehall II and atherosclerosis risk in communities studies. Alzheimers Dement. doi: 10.1002/alz.12419.Online ahead of print. 10.1002/alz.12419 PMC9292245 34338426

[iyab185-B20] Lindeboom J , Weinstein H. 2004. Neuropsychology of cognitive ageing, minimal cognitive impairment, Alzheimer’s disease, and vascular cognitive impairment. Eur J Pharmacol. 490:83–86. 15094075 10.1016/j.ejphar.2004.02.046

[iyab185-B21] Malek-Ahmadi M , Su Y, Jansen WJ. 2021. Vascular factors and vascular lesions in pre-clinical Alzheimer’s disease. Front Neurol. 12:738465. 34539565 10.3389/fneur.2021.738465 PMC8442912

[iyab185-B22] Mayeux R , Stern Y. 2012. Epidemiology of Alzheimer disease. Cold Spring Harb Perspect Med. 2(8):a006239. 10.1101/cshperspect.a006239 PMC3405821 22908189

[iyab185-B23] Miller MB , Reed HC, Walsh CA. 2021. Brain somatic mutation in aging and Alzheimer’s disease. Annu Rev Genomics Hum Genet. 22:239–256. 33979534 10.1146/annurev-genom-121520-081242 PMC8612367

[iyab185-B152] Milind N , Preuss C, Haber A, Ananda G, Mukherjee S. et al Transcriptomic stratification of late-onset Alzheimer’s cases reveals novel genetic modifiers of disease pathology. P LoS Genet. 2020 Jun 3;16:e1008775. doi: 10.1371/journal.pgen.1008775. P MID: 32492070; P MCID: P MC7295244. 10.1371/journal.pgen.1008775 PMC7295244 32492070

[iyab185-B24] Mirra SS , Hart MN, Terry RD. 1993. Making the diagnosis of Alzheimer’s disease. A primer for practicing pathologists. Arch Pathol Lab Med. 117:132–144. 8427562

[iyab185-B25] Montine TJ , Phelps CH, Beach TG, Bigio EH, Cairns NJ, et al; Alzheimer’s Association. 2012. National Institute on Aging-Alzheimer’s Association guidelines for the neuropathologic assessment of Alzheimer’s disease: a practical approach. Acta Neuropathol. 123:1–11. 22101365 10.1007/s00401-011-0910-3 PMC3268003

[iyab185-B151] Monterey MD , Wei H, Wu X, Wu JQ. The Many Faces of Astrocytes in Alzheimer’s Disease. Front Neurol. 2021 Aug 31;12:619626. doi:10.3389/fneur.2021.619626. P MID: 34531807; P MCID: P MC8438135. 10.3389/fneur.2021.619626 PMC8438135 34531807

[iyab185-B26] Mukherjee S , Mez J, Trittschuh EH, Saykin AJ, Gibbons LE, et al; EPAD Study Group. 2020. Genetic data and cognitively defined late-onset Alzheimer’s disease subgroups. Mol Psychiatry. 25:2942–2951. 30514930 10.1038/s41380-018-0298-8 PMC6548676

[iyab185-B27] Mullane K , Williams M. 2020. Alzheimer’s disease beyond amyloid: can the repetitive failures of amyloid-targeted therapeutics inform future approaches to dementia drug discovery? Biochem Pharmacol. 177:113945. 32247851 10.1016/j.bcp.2020.113945

[iyab185-B28] Mullard A. 2021. Landmark Alzheimer’s drug approval confounds research community. Nature. 594:309–310. 34103732 10.1038/d41586-021-01546-2

[iyab185-B29] National Institute On Aging Reagan Institute Working Group. 1997. Consensus recommendations for the postmortem diagnosis of Alzheimer’s disease. National Institute on Aging and Reagan Institute Working Group on Diagnostic Criteria for the Neuropathological Assessment of Alzheimer’s Disease. Neurobiol Aging. 18(4 Suppl):S1–S2. 9330978

[iyab185-B30] Neff RA , Wang M, Vatansever S, Guo L, Ming C, et al 2021. Molecular subtyping of Alzheimer’s disease using RNA sequencing data reveals novel mechanisms and targets. Sci Adv. 7:eabb5398. 10.1126/sciadv.abb5398 PMC7787497 33523961

[iyab185-B31] Nelson PT , Braak H, Markesbery WR. 2009. Neuropathology and cognitive impairment in Alzheimer disease: a complex but coherent relationship. J Neuropathol Exp Neurol. 68:1–14. 19104448 10.1097/NEN.0b013e3181919a48 PMC2692822

[iyab185-B32] Nestler EJ , Hyman SE. 2010. Animal models of neuropsychiatric disorders. Nat Neurosci. 13:1161–1169. 20877280 10.1038/nn.2647 PMC3750731

[iyab185-B33] Neuner SM , Heuer SE, Huentelman MJ, O'Connell KMS, Kaczorowski CC. 2019. Harnessing genetic complexity to enhance translatability of Alzheimer’s disease mouse models: a path toward precision medicine. Neuron. 101:399–411.e5. 30595332 10.1016/j.neuron.2018.11.040 PMC6886697

[iyab185-B34] Oblak AL , Forner S, Territo PR, Sasner M, Carter GW, et al 2020. Model organism development and evaluation for late-onset Alzheimer’s disease: MODEL-AD. Alzheimers Dement (NY). 6:e12110. 10.1002/trc2.12110 PMC7683958 33283040

[iyab185-B35] Oddo S , Caccamo A, Shepherd JD, Murphy MP, Golde TE, et al 2003. Triple-transgenic model of Alzheimer’s disease with plaques and tangles: intracellular Abeta and synaptic dysfunction. Neuron. 39:409–421. 12895417 10.1016/s0896-6273(03)00434-3

[iyab185-B36] Ou YN , Zhu JX, Hou XH, Shen XN, Xu W, Dong Q, et al 2020. Associations of infectious agents with Alzheimer’s disease: a systematic review and meta-analysis. J Alzheimers Dis. 75:299–309. 32280095 10.3233/JAD-191337

[iyab185-B37] Pande M , Srivastava R. 2019. Molecular and clinical insights into protein misfolding and associated amyloidosis. Eur J Med Chem. 184:111753. 31622853 10.1016/j.ejmech.2019.111753

[iyab185-B38] Puzzo D , Gulisano W, Palmeri A, Arancio O. 2015. Rodent models for Alzheimer’s disease drug discovery. Expert Opin Drug Discov. 10:703–711. 25927677 10.1517/17460441.2015.1041913 PMC4592281

[iyab185-B39] Ryan NS , Rossor MN, Fox NC. 2015. Alzheimer’s disease in the 100 years since Alzheimer’s death. Brain. 138:3816–3821. 26541346 10.1093/brain/awv316

[iyab185-B40] Santayana G. 1905. Reason in common sense. In: The Life of Reason or the Phases of Human Progress. New York: Prometheus Books, p. 82.

[iyab185-B41] Selkoe DJ , Podlisny MB, Joachim CL, Vickers EA, Lee G, et al 1988. Beta-amyloid precursor protein of Alzheimer disease occurs as 110- to 135-kilodalton membrane-associated proteins in neural and nonneural tissues. Proc Natl Acad Sci USA. 85:7341–7345. 3140239 10.1073/pnas.85.19.7341 PMC282182

[iyab185-B42] Sen A , Akinola M, Tai XY, Symmonds M, Davis Jones G, et al 2021. An investigation of levetiracetam in Alzheimer's disease (ILIAD): a double-blind, placebo-controlled, randomised crossover proof of concept study. Trials. 22:508. 34332638 10.1186/s13063-021-05404-4 PMC8325256

[iyab185-B43] Serpell L. 2014. Amyloid structure. Essays Biochem. 56:1–10. 25131583 10.1042/bse0560001

[iyab185-B44] Tagai K , Ono M, Kubota M, Kitamura S, Takahata K, et al 2021. High-contrast in vivo imaging of tau pathologies in Alzheimer’s and non-Alzheimer’s disease tauopathies. Neuron. 109:42–58.e8. 33125873 10.1016/j.neuron.2020.09.042

[iyab185-B45] Tanzi RE , Kovacs DM, Kim TW, Moir RD, Guenette SY, et al 1996. The gene defects responsible for familial Alzheimer’s disease. Neurobiol Dis. 3:159–168. 8980016 10.1006/nbdi.1996.0016

[iyab185-B46] Tariot PN , Lopera F, Langbaum JB, Thomas RG, Hendrix S, et al; Alzheimer’s Prevention Initiative. 2018. The Alzheimer’s prevention initiative autosomal-dominant Alzheimer's disease trial: a study of crenezumab versus placebo in preclinical *PSEN1* E280A mutation carriers to evaluate efficacy and safety in the treatment of autosomal-dominant Alzheimer's disease, including a placebo-treated noncarrier cohort. Alzheimers Dement (N Y). 4:150–160. 29955659 10.1016/j.trci.2018.02.002 PMC6021543

[iyab185-B47] Trudler D , Nazor KL, Eisele YS, Grabauskas T, Dolatabadi N, et al 2021. Soluble alpha-synuclein-antibody complexes activate the NLRP3 inflammasome in hiPSC-derived microglia. Proc Natl Acad Sci USA. 118:e2025847118. 33833060 10.1073/pnas.2025847118 PMC8054017

[iyab185-B48] Vina J , Sanz-Ros J. 2018. Alzheimer’s disease: only prevention makes sense. Eur J Clin Invest. 48:e13005. 30028503 10.1111/eci.13005

[iyab185-B49] Vogel JW , Young AL, Oxtoby NP, Smith R, Ossenkoppele R, et al; Alzheimer’s Disease Neuroimaging Initiative. 2021. Four distinct trajectories of tau deposition identified in Alzheimer’s disease. Nat Med. 27:871–881. 33927414 10.1038/s41591-021-01309-6 PMC8686688

[iyab185-B50] Wegmann S , Biernat J, Mandelkow E. 2021. A current view on tau protein phosphorylation in Alzheimer’s disease. Curr Opin Neurobiol. 69:131–138. 33892381 10.1016/j.conb.2021.03.003

[iyab185-B51] Wolfe MS. 2012. Processive proteolysis by gamma-secretase and the mechanism of Alzheimer’s disease. Biol Chem. 393:899–905. 22944690 10.1515/hsz-2012-0140

[iyab185-B52] Yankner BA , Mesulam MM. 1991. Beta-amyloid and the pathogenesis of Alzheimer’s disease. N Engl J Med. 325:1849–1857. 1961223 10.1056/NEJM199112263252605

